# Biological properties of bone marrow stem cells and adipose-derived stem cells derived from T2DM rats: a comparative study

**DOI:** 10.1186/s13578-020-00465-5

**Published:** 2020-09-07

**Authors:** Lei Wang, Shaojie Shi, Ruiping Bai, Yue Wang, Zhao Guo, Doudou Li

**Affiliations:** 1 Key Laboratory of Shaanxi Province for Craniofacial Precision Medicine Research, College of Stomatology, Xi’an Jiaotong University, 98 XiWu Road, Xi’an, 710004 Shaanxi People’s Republic of China; 2 Clinical Research Center of Shaanxi Province for Dental and Maxillofacial Diseases, College of Stomatology, Xi’an Jiaotong University, Xi’an, 710004 Shaanxi People’s Republic of China; 3 State Key Laboratory of Military Stomatology & National Clinical Research Center for Oral Diseases & Shaanxi Engineering Research Center for Dental Materials and Advanced Manufacture, School of Stomatology, Air Force Medical University, Xi’an, Shaanxi People’s Republic of China; 4 Department of Math and Physics, School of Basic Medicine, Air Force Medical University, Xi’an, 710032 Shaanxi People’s Republic of China

**Keywords:** Type 2 diabetes mellitus, Mesenchymal stem cells, Cell proliferation, Osteogenic induction, Cell sheets

## Abstract

**Background:**

Patients with type 2 diabetes mellitus (T2DM), especially those with poor glycemic control, are characterized by low bone mass and destruction of bone microstructure. Nowadays, autologous mesenchymal stem cells (auto-MSCs) have been used to repair defects and promote tissue regeneration due to handy source, low immunogenicity and self-renewing and multi-differentiating potential. However, T2DM changed the biological properties of auto-MSCs, and investigating the most suitable auto-MSCs for T2DM patients becomes a focus in tissue engineering.

**Results:**

In this research, we compared the biological characteristics of adipose-derived stem cells (ASCs) and bone marrow stem cells (BMSCs) derived from T2DM rats. These results demonstrated that ASCs had a higher proliferation rate, colony-formation and cell-sheet forming ability, while BMSCs got better osteogenesis-related staining, expression of osteogenesis-related genes and proteins, and osteogenic capacity in vitro.

**Conclusions:**

As it turned out, ASCs from T2DM had a higher proliferation, while BMSCs had significantly higher osteogenetic ability no matter in vitro and in vivo. Therefore, we should take into account the specific and dominated properties of MSC according to different needs to optimize the protocols and improve clinical outcomes for tissue regeneration of T2DM patients.

## Introduction

Diabetes mellitus, characterized by high glucose levels, is a chronic metabolic disorder, the incidence of which ranks second only after cardiovascular disease. It could be categorized into type 1 diabetes mellitus (T1DM) and T2DM, of which T2DM accounts for 90–95% [1]. Diabetic osteoporosis, characterized with low bone mass and destruction of bone microstructure, is adverse for diabetes patients due to hyperglycemic toxicity, abnormal insulin and cytokine levels as well as oxidative stress [2, 3]. The fracture risk increased and the fracture healing decreased significantly at the same time in diabetic patients [4]. The proliferation and osteogenesis of alveolar bone-derived osteoblasts from T2DM were impaired [5]. Patients with poor glycemic control had inferior osseointegration of dental implants [6, 7], compromised wound healing process in the early stage [8].

In recent years, the discovery of stem cells has prompted a number of preclinical trials in tissue engineering. Stem cells could repair and even regenerate damaged tissues and organs via accelerating wound closure, increasing angiogenesis and regulating extracellular matrix remodeling [9]. Among them, mesenchymal stem cells (MSCs) are more applicable for therapeutic exploration in response to easy clinical availability, relatively simple culture and stable differentiation ability. For instance, it has been applied to improve the osteogenesis of diabetic patients [10]. Thereinto, BMSCs and ASCs are most commonly used for regenerative therapies.

However, assays to apply and modify MSCs are mostly based on the acquisition of allogeneic stem cells, which is limited in clinical application [10]. Meantime, some studies describing the generation of antibodies in host against graft and provoking immune rejection of allogeneic MSCs (allo-MSCs) suggest it may not be hypoimmunogenic [11]. By contrast, auto-MSCs appear more extensive application prospects owning to clinical availability, long-term survival and transplanted tolerance. ASCs represent a promising option with a wide range of sources, easy to obtain and culture and suffer little. BMSCs are difficult to obtain and costly, but it is undeniable that BMSCs own better osteogenic differentiation. However, the number, proliferation, survival ability and osteogenic differentiation of BMSC from T2DM are significantly lower than in non-diabetic individuals [12–14]. By contrast, the number of ASCs from diabetic individuals did not decrease, and the growth curve was comparable to that of the control group [15].

For patients with T2DM, the biological properties of autologous stem cells are distinct from healthy people owing to the changes in their microenvironment [16, 17]. However, there is no much discussion about the comparison between auto-ASCs and auto-BMSCs from T2DM. How to select the most suitable autologous stem cells in T2DM patients for tissue regeneration has also become a research hotspot. In this study, the basic biological characteristics of ASCs and BMSCs from T2DM rats were compared, so as to clarify the mechanisms involved and provide guidance for future studies.

## Materials and methods

### Induction of type II diabetics rat models

24 male SD rats (8 weeks, 150–200 g), provided by the Experimental Animal Center (Accreditation No SCXK 2014-002), were applied for this study in compliance with Ethical Norms (Ethical Accreditation No 2017 kq-025). The rats were randomly divided into the T2DM group (n = 16) and the control group (n = 8). T2DM rats were induced by feeding with high fat and high sugar diet (basic feed, 69.5%; sucrose, 10%; egg yolk power, 10%; cholesterol, 0.5%; fat, 10%), and intraperitoneal single injection of 35 m/kg STZ (Sigma, USA) after 4 weeks feeding [1, 18], while control group received injection of citric acid buffer (Solarbio, China) at a dose of 35 mg/kg combining with normal forage feeding. A week later, blood samples from tail tips were taken in order to measure the glucose value and monitored weekly. Rats with random blood glucose above 300 mg/dL (16.7 mmol/L) for 4 weeks were considered to be successful models and selected for the subsequent research.

### ASCs and BMSCs isolation and culture from T2DM rats

T2DM rats were sacrificed. Then, the adipose tissue in the groin was taken, cut into pieces and digested with 0.2% collagenase I (Gibco, USA) for 1 h. The solution was filtered through 200 mesh screen, centrifuged (1000 r/min, 5 min) and then resuspended with a-MEM complete medium (10% serum [BI, Israel], 1% Penicillin–Streptomycin Solution [HyClone, USA], 89% basic medium [HyClone, USA]). Cells were eventually plated in T75 flasks (the cells extracted from a rat was inoculated in a T75 flask) and incubated until it reached 80% confluence. After that, replace the medium every 3 days and passage it until passage 3.

In the process of adipose tissue digestion, the bilateral lower limb bones were obtained. Marrow cavity was rinsed repeatedly with complete medium until it became white. The suspension was collected, centrifuged (1000 r/min, 5 min) and resuspension. Cells were plated in T75 flasks (the cells extracted from a rat was inoculated in a T75 flask), cultured in an incubator and passaged until at passage 3 to be used in the next experiments.

### Identification of stem cell surface markers

ASCs and BMSCs of P3 were washed with sterile PBS for 3 times, digested with trypsin and centrifuged (1000 r/min, 5 min). Then the complete medium was used to resuspend the cell. The cell suspension was split into 1.5 ml centrifuge tubes, and the number of cells was controlled with 5 × 10^5^–1 × 10^6^ in each tube. Antibodies (CD29, CD90, CD34 and CD45 [CST, USA]) were added, and the expression level of molecules on the cell surface was detected by flow cytometry [BD, USA].

### Colony-forming assay and Cell proliferation analysis

ASCs and BMSCs were plated in the 100 mm culture dish at a density of 1000/dish. Then fixed them with formaldehyde after 10 days. 0.1% crystal violet dye (Solaibio, China) was used to stain cells for 15 min. After removing float dyestuff, the clone formation was observed and counted under the microscope. A colony containing more than 50 cells was considered a clone (Cloning formation rate = Number of clone/Number of original inoculated cells × 100%).

A portion of the ASCs and BMSCs was used to estimate cell amplification potential. They were inoculated on 96-well plates at a density of 3000/well and set as one group per day. Complete medium and CCK-8 solution (EnoGene, China) was added to the test groups in a ratio of 10:1 with mixture 110 uL at the same time every day. Then it was incubated in an incubator for 3 h. Detect the absorbance value (OD value) at 450 nm. After 8 days of operation, the growth curve was drawn depending on the results.

### Adipogenic and osteogenetic differentiation induction

ASCs and BMSCs of P3 were plated on 6-well plates at a density of 2.5 × 10^5^/well with lipogenic induction medium (1 μM dexamethasone, 200 μM Indometacin, 0.5 mM Isobutylmethylxanthine, 10 μg/ml Insulin [MP, USA]). After 14 days, samples were fixed with polyformaldehyde and stained with oil red O solution (Sigma, USA). The staining results were observed under the microscope.

Simultaneously, both cells were plated at the same density with osteogenic induction medium (10 mM β-glycerophosphate, 50 mg/mL ascorbic acid, and 10^−7^M dexamethasone [MP, USA]). After 7 and 28 days, samples were fixed and then stained with BCIP/NBT dye (CWBIO, China), alizarin red dye (Solaibio, China) for ALP production and ECM mineralization respectively. We used ImageJ to select multiple fields and compare the ratio occupied by staining areas in the total field of view for ALP semi-quantitation. Acetylpyridine chloride eluent was added in every well with 1 mL/well and then measured the OD value at 620 nm for quantitative analysis of alizarin red staining.

### Real-time quantitative polymerase chain reaction (qPCR) and Western Blot analysis

The BMSCs and ASCs were induced with osteogenic induction medium for 7 days. Whereafter, Total RNA was extracted, quantified and reverse transcribed into complementary DNA (cDNA). qPCR was applied to detect the relative expression of osteogenic genes (runt-related transcription factor 2 (*RUNX2*), alkaline phosphatase (*ALP*), collagen type I (*COL1*), bone morphogenetic protein (*BMP*), using TB Green^®^ Premix Ex Taq™ II kit (Takara, Japan). Setting β-actin as the endogenous reference, the relative expression of osteogenic gene was obtained by calculating the CT value. The primers sequences are listed in Table 1.

**Table 1:** Primers sequences for qPCR

Gene	Forward primer sequence (5′-3′)	Reverse primer sequence (5′-3′)
*β*-*actin*	TGGCACCCAGCACAATGAA	CTAAGTCATAGTCCGCCTAGAAGCA
*RUNX2*	CCATAACGGTCTTCACAAATCCT	TCTGTCTGTGCCTTCTTGGTTC
*ALP*	CCTTGTAGCCAGGCCCATTG	GGACCATTCCCACGTCTTCAC
*COL1*	GCCTCCCAGAACATCACCTA	GCAGGGACTTCTTGAGGTTG
*BMP*	CAACACCGTGCTCAGCTTC C	TTCCCACTCATTTCTGAAAGTTCC

*RUNX2* runt-related transcription factor 2, *ALP* alkaline phosphatase, *COL1* collagen type I, *BMP* bone morphogenetic protein

Meanwhile, the total protein of both samples was extracted with PIPA lysate (Jingcai, China) after osteogenic induction for 7 days and performed protein quantification. Rapid gel kit (Jingcai, China) was used for protein electrophoresis and then the proteins were transferred to the polyvinylidene fluoride membrane (Solaibio, China). After blocking with skimmed milk powder (BD, USA), membranes were incubated with primary antibody (Anti-GAPDH antibody, Anti-ALP antibody, Anti-RUNX2 antibody, Anti-OCN antibody [CST, USA]) overnight at 4°Cand secondary antibody (KangWei, China) at room temperature for 1 h. Ultimately, the photoluminescence was developed by utilizing Genshare supersensitive luminescent substrate detection kit (Jingcai, China), and the gel image analysis was carried out.

### Cell-sheet formation and morphologic observation

The two type cells were inoculated on 6-well plates until confluent to 90% with conventional medium and then changed into cell-sheets induction medium (containing 50 mg/ml ascorbic acid). After 10 days, the cell-sheets was formed and then fixed with 4% paraformaldehyde (Solaibio, China). Surface and cross-sectional morphology of cell-sheets were detected by Electron Microscopy. At the same time, histological sections and H&E staining were carried out. The thickness of cell-sheets was measured by Adobe Photoshop CC 2018.

### Animal groups and surgery

T2DM rats (n = 16) were operated with sterile conditions under anesthesia. Following disinfection and towel laying, an incision about 2 cm in length was made in the middle of the cranial apex to expose the parietal bone. Subsequently, a defect with diameter of 5 mm was formed using a trephine and circular grinding drill between the coronal suture and the herringbone suture. Afterward, both cells (P3, the same number of two types of cells) were acquired and resuspended with medium (200 μL). The suspensions were mixed with Bio-oss^®^ bone powder (Geistlich, Switzerland) and placed in an incubator for 30 min. Then, compounds were implanted into the defect site of T2DM rats (each group: n = 8). At 6- and 12-week after operation, the specimens were collected and fixed with paraformaldehyde for CT scanning. The bone defect healing was analyzed after 3D reconstruction. A cylinder with a diameter of 5 mm and a thickness of 1 mm was selected as the area of interest (ROI) to be analyzed. CT values between 700 and 2000 were defined as new bone, shown in green. CT value above 2000 was defined as bone powder, shown in red. Bone Volume/Total Volume (BV/TV), Trabecular thickness (Tb.Th) and trabecular separation (Tb.Sp) in the ROI were analyzed.

Then, specimens were decalcified with 17% EDTA (PuZhen, China) for 20-30 days. The fluid was changed every 3 days until the bone tissue became soft. The samples were then dehydrated, transparent, waxed, embedded and sliced. Sections were dewaxed with xylene for 5–10 min, then immersed in alcohol of 100% to low concentration for 3–5 min, then slightly washed with distilled water. The sections were dyed with Hematoxylin (Feiyang, China) for 10–20 min and rinsed by running water for 5 min until the sections turned blue. The sections were differentiated in 1% alcohol hydrochloride for a few seconds until the color became light. After running water rinsed, distilled water rinsed sections again. Immerse them in 70% and 80% alcohol for 3 min for dehydration. Redye them with Eosin (Feiyang, China) for 2 min. Low concentration to 100% gradient alcohol were used to dehydrate, then immersed them into xylene (Feiyang, China) for transparent. Finally, neutral gum (Feiyang, China) was to seal sections, and observed section panorama under the microscope and take photos.

### Statistical analyses

All values were reported as mean ± SD. Statistical analysis was performed using SPSS software (version17.0). Unrelated *t* test was used for comparison between the two groups for significant differences in the repeated measure. The degree of statistical significance was considered P < 0.05.

## Results

### Type II diabetic rat model

After 4 weeks of specialty diet, T2DM rats appeared moderate weight gain compared with control group. After 1 week of injection, T2DM rats showed obvious symptoms of polyphagia, polydipsia and polyuria. Blood glucose of T2DM group remained stable and more than 16.7 mmol/L, while control group remained normoglycaemic (Table 2). Whereafter, T2DM rats exhibited signs of wasting gradually and got worse, and their weight was much lower than that of normal group. The two groups have significant differences between blood glucose and body weight (P < 0.01).

**Table 2:** Comparison of blood glucose values between diabetic and normal rats (mmol/L, x ±  ®s)

Group	Before	1 W	2 W	3 W	4 W
T2DM	6.58 ± 0.100	27.39 ± 1.504	28.30 ± 1.105	28.58 ± 0.725	30.09 ± 0.293
Control	6.76 ± 0.038	6.61 ± 0.425	6.50 ± 0.260	6.89 ± 0.150	6.51 ± 0.278

*T2DM* type2 diabetes mellitus, *W* week

### Expression of stem cell surface markers

**Fig. 1:**
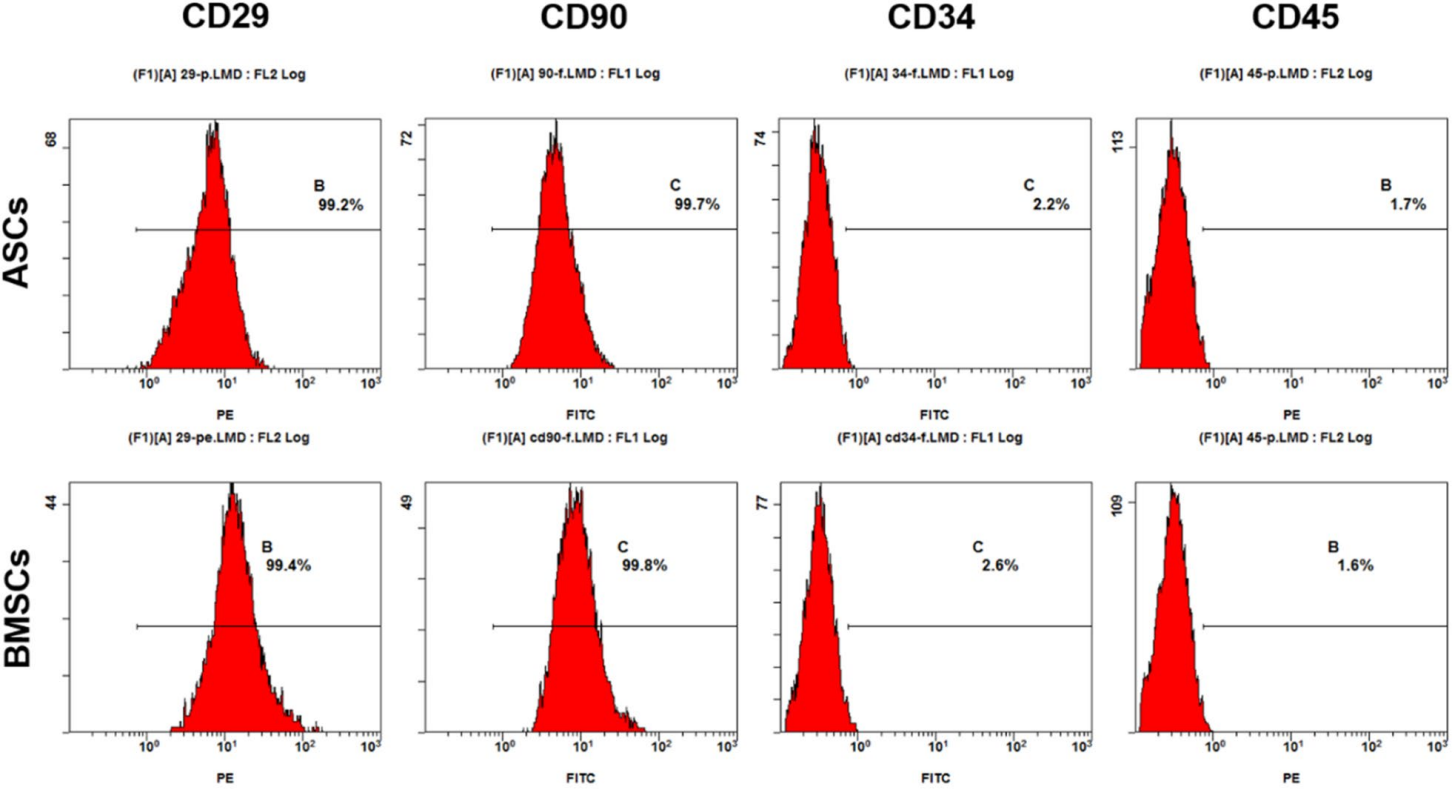
Expression of ASCs and BMSCs surface markers

Results showed that both cells positively expressed stem cell related markers (CD29 and CD90), but not blood cell related markers (CD34 and CD45). The expression rates of CD29, CD90, CD34 and CD45 were 99.2%, 99.7%, 2.2% and 1.7% respectively in ASCs, and 99.4%, 99.8%, 2.6% and 1.6% respectively in BMSCs.There was no significant statistical difference in the expression levels of the stem cell related molecules (P > 0.05) (Fig. 1).

### Colony-forming units (CFU) and cell growth curve determination

**Fig. 2:**
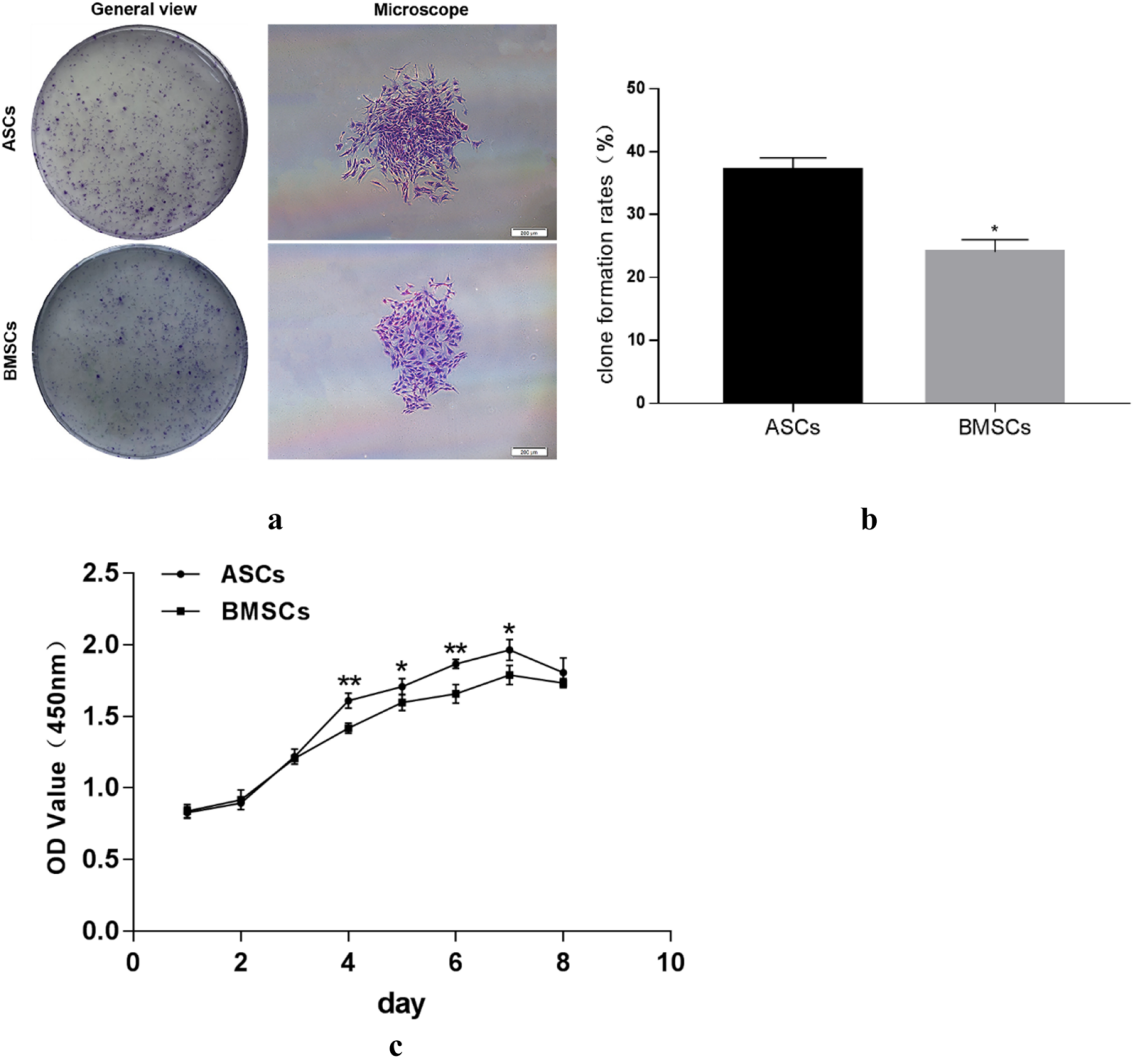
**a** General and microscope view of ASCs and BMSCs clone formation. **b** Quantitative comparison between ASCs and BMSCs Clone formation rate. **c** Growth curves of ASCs and BMSCs. **a** scale bar = 200 μm; *P < 0.05; **P < 0.01

The 10th day after inoculation, it was found by crystal violet staining that a large number of cell clones were formed and the cells clustered to form a colony (Fig. 2a). There was a statistical difference in CFU rate between BMSCs and ASCs, while clone formation rates of ASCs and BMSCs were 35.67% and 25.67% respectively (Fig. 2b).

At the same time, the quantity growth trend of both cells tended to form a significant S-shaped growth curve. The number of cells remained practically unchanged on the first day, which was defined as the retention phase. From the second day, it entered the logarithmic growth phase and reached the plateau on the 7th day. The proliferation rate appeared dramatically different from the third Day. More precisely, the OD value of ASCs was gradually higher than BMSCs, and the difference was statistically significant on day 4, 5, 6 and 7 (p < 0.05) (Fig. 2c).

### Adipogenesis and Osteogenesis staining

**Fig. 3:**
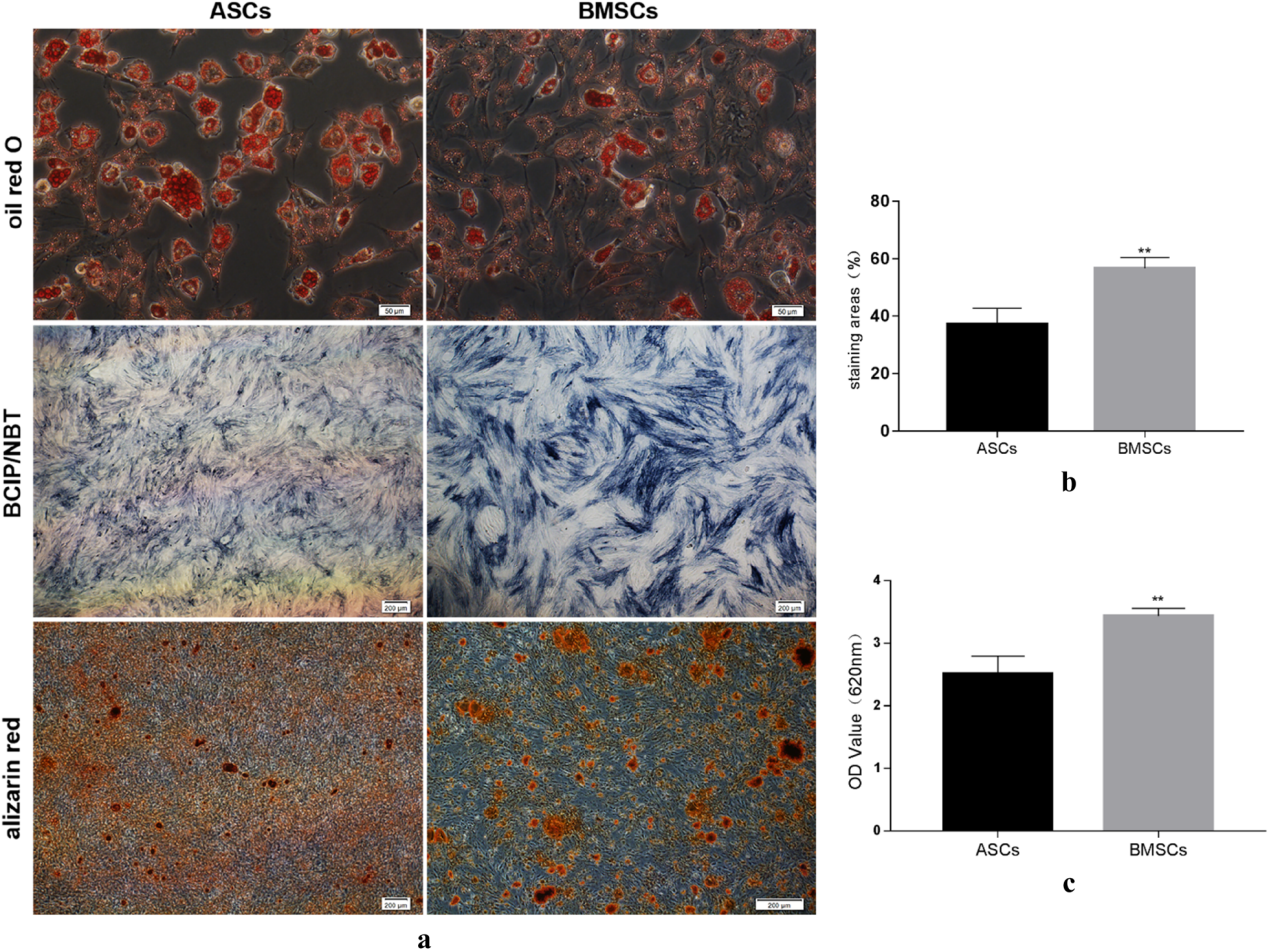
Observation of Osteogenesis and adipogenesis staining of ASCs and BMSCs. **a** Oil red staining for lipid droplets after 14-day lipogenic differentiation; BCIP/NBT staining for alkaline phosphatase after 7-day osteogenic differentiation; Alizarin red staining for mineralized nodules after 28-day osteogenic differentiation of ASCs and BMSCs. **b** Semi-quantitative comparison of BCIP/NBT staining using Image J software between ASCs and BMSCs. **c** Semi-quantitative comparison of Alizarin red staining using spectrophotometer at 620 nm between ASCs and BMSCs. **a** Oil red staining, scale bar = 50 μm; BCIP/NBT staining, scale bar = 200 μm; Alizarin red staining, scale bar = 200 μm. *P < 0.05; **P < 0.01

Both cells could produce lipid droplets and even fused to form beads. The number and volume of fat droplets of ASCs were more and larger than BMSCs (Fig. 3a). It confirmed that ASCs had better adipogenic differentiation than BMSCs.

The two groups were stained blue-purple with BCIP/NBT dye after 7 days of osteogenic induction. And the expression of ALP in BMSCs was significantly higher than that in ASCs (Fig. 3a). Semi-quantitation showed that BMSCs presented better early osteogenesis (Fig. 3b). The mineralized nodules in the cells were shown by alizarin red staining, and these results demonstrated that BMSCs had more mineralized nodules and even connected into small pieces compared with ASCs (Fig. 3a). The semi-quantitative analysis showed that the calcium nodules in BMSCs were significantly higher than those in ASCs group (p < 0.01) (Fig. 3c).

### Osteogenic-related gene and protein expression

**Fig. 4:**
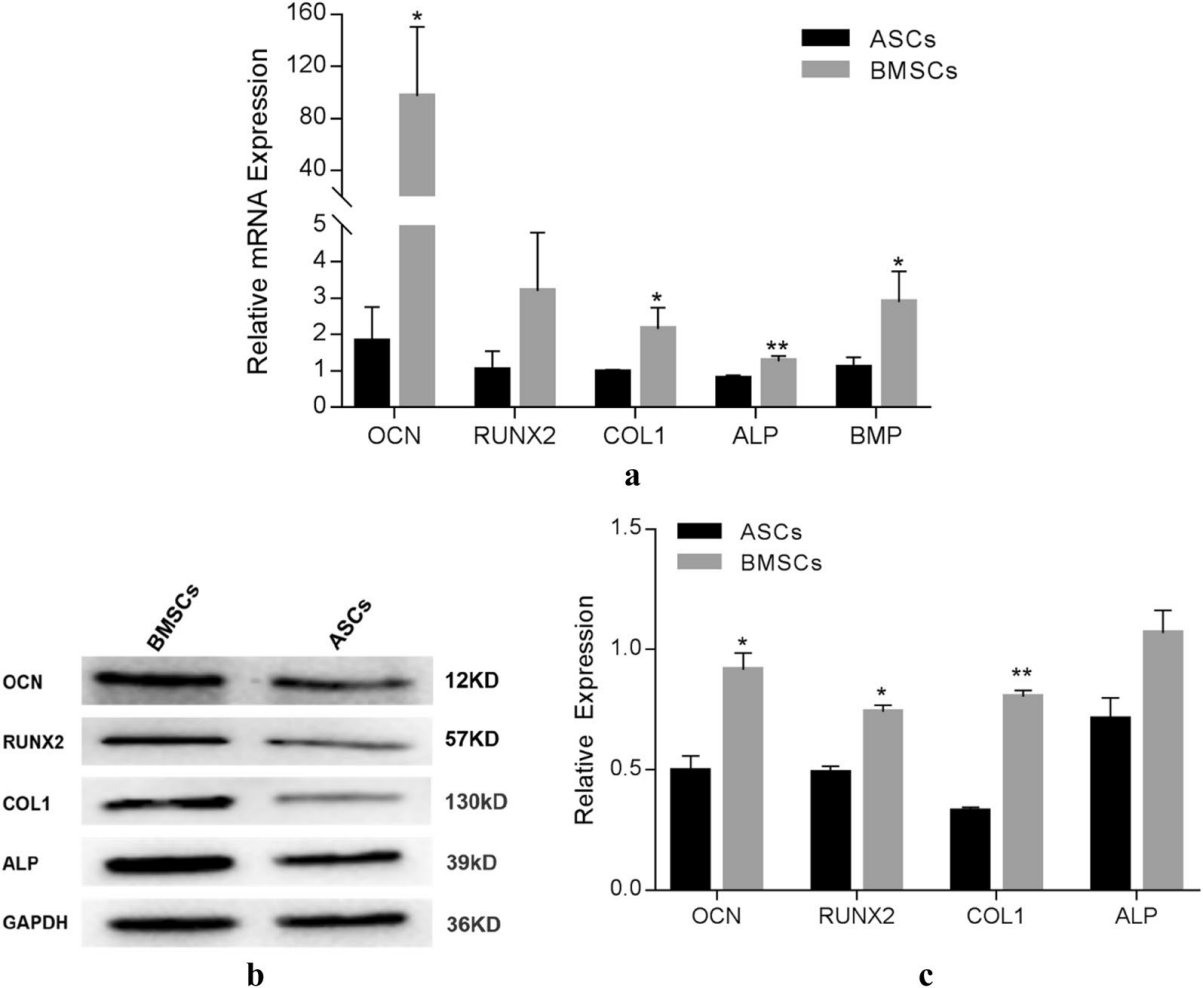
Expression of osteoblast-related genes and proteins of ASCs and BMSCs. **a** Relative mRNA expression level of osteoblast-related genes in BMSCs and ASCs after 7-days osteogenic induction. **b** The relative protein level in BMSCs and ASCs after 7-days osteogenic induction. **c** Quantitative comparison of the relative protein level. Notes: *P < 0.05; **P < 0.01. Abbreviations: *RUNX2*, runt-related transcription factor 2; *ALP*, alkaline phosphatase; *OCN*, osteocalcin; *BMP*, bone morphogenetic protein; *GAPDH*, glyceraldehyde-3-phosphate dehydrogenase; mRNA, messenger RNA

The results of qPCR showed that the mRNA expression levels of *ALP*, *COL1*, and *BMP* in BMSC were significantly higher than those in ASCs (*COL1*:p < 0.05; *BMP*: p < 0.05; *ALP*: p < 0.01), especially *OCN* was almost 50-fold in BMSCs than ASCs (P < 0.05), whereas *RUNX2* expression level in BMSCs was little higher than ASCs, but not statistically significant (Fig. 4a).

The osteogenic protein expression was analyzed by western blotting. In agreement with the qPCR result, all the detected osteogenic proteins in BMSCs were higher than ASCs (Fig. 4b), especially protein expression level of OCN, RUNX2, COL1 in BMSCs is almost 1.5-2 times higher than in the ASCs (Fig. 4c), with a significant difference between both of them.

### Cell-sheet morphologic observation and comparison

**Fig. 5:**
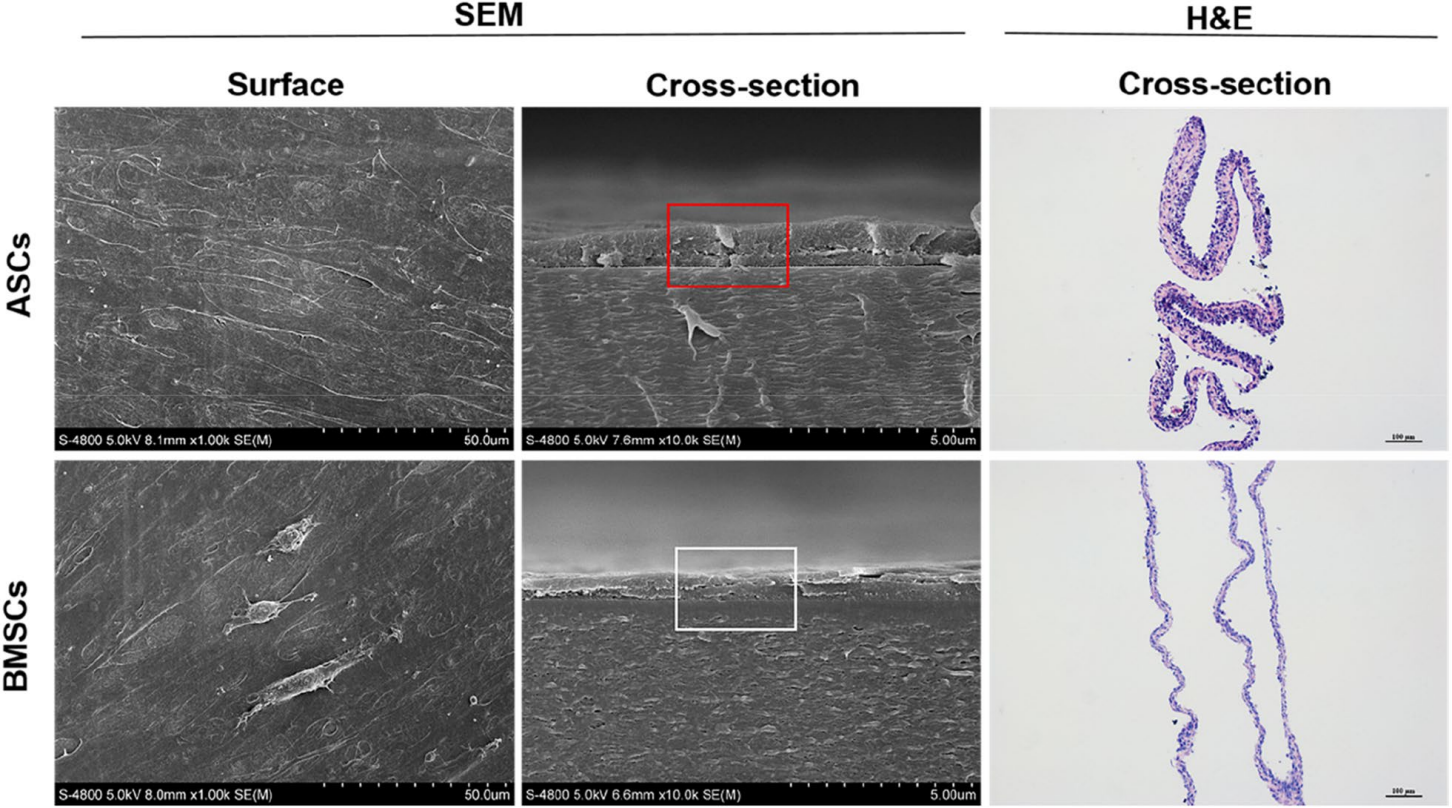
Observation of cell-sheets morphology by Scanning Electron Microscopy of ASCs and BMSCs; HE staining of cell sheets of ASCs and BMSCs. Note: SEM, surface view, × 1.00 K; Cross-section view, × 10.0 K. H&E, Cross-section view, scale bar = 100 μm. Abbreviations: SEM, scanning electron microscope; H&E, hematoxylin-eosin

Both cells could form a large amount of extracellular matrix, ultimately connected into sheets after 7-days induction. Under Scanning Electron Microscopy, the cells were joined to each other by a platelike pseudopod in surface view. The ASCs cell-sheet was significantly thicker than BMSCs in cross-section (p < 0.05) (Fig. 5). H&E staining showed that both type cells formed a cell-stroma-cell sandwich structure, and ASCs had more cell layers and extracellular matrix, indicating that ASCs had stronger proliferation capacity than BMSC, even maybe the more remarkable secreting of extracellular matrix (Fig. 5).

### Micro CT scanning histological sections and H&E staining and Histological analysis

**Fig. 6:**
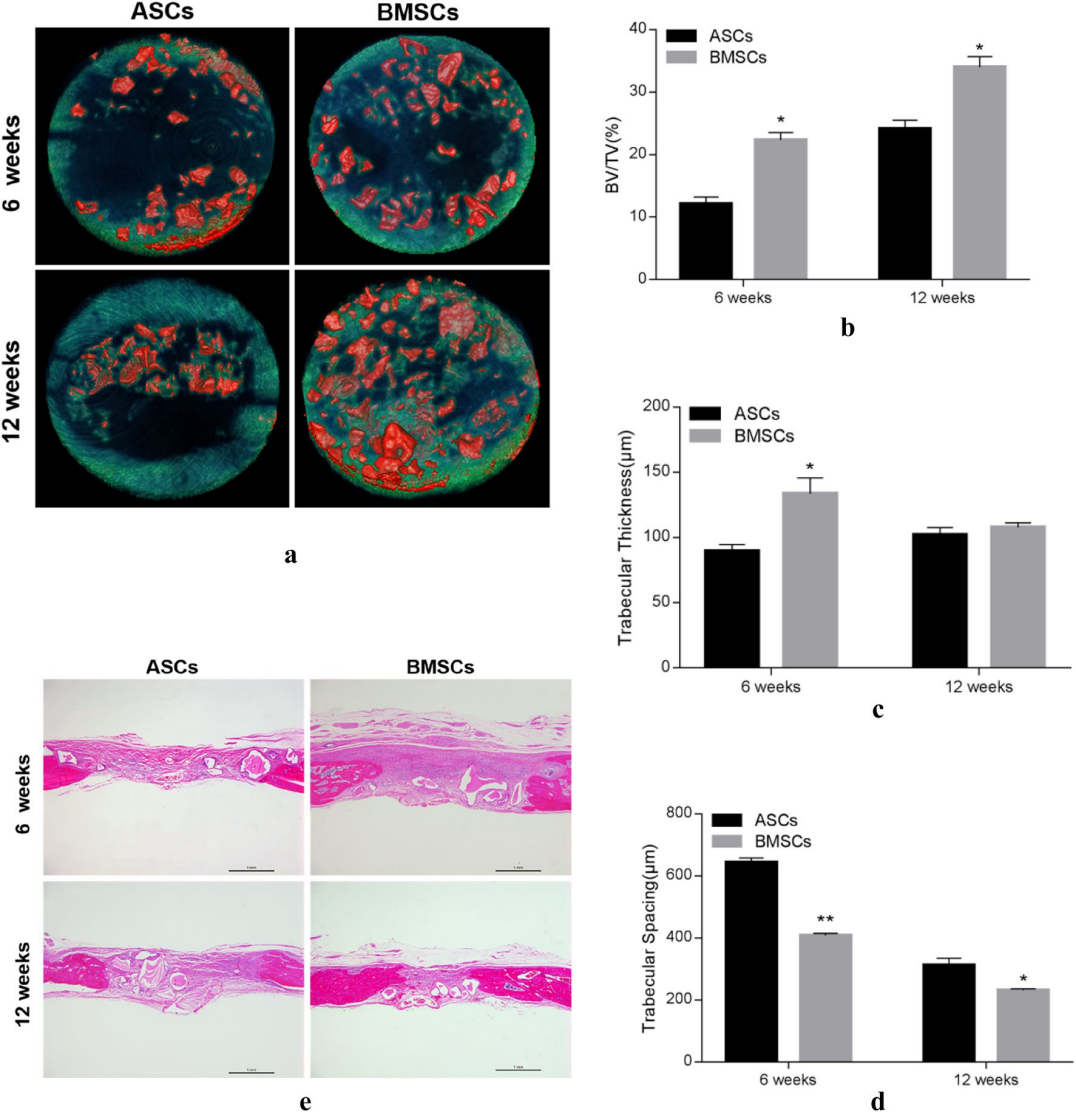
Micro-CT scanning and quantitative analysis and H&E staining at the 6, 12 weeks. **a** Bone defect models with ASCs/BMSCs-Bioss powder complex were scanned by micro-CT at 6 weeks, 12 weeks postimplantation. Quantitative analysis of regenerated bone of both groups was evaluated in BV/TV (**b**), Tb. Th (**c**) and Tb.Sp (**d**) (*p < 0.05, **p < 0.01) (**e**) Histological analysis was performed by slicing followed by H&E staining after 6 and 12 weeks of healing bone defect models with ASCs/BMSCs-Bioss powder complex. CT, computed tomography; Tb.Th, trabecular thickness; Tb.Sp, trabecular separation; BV/TV, bone volume to tissue volume; w, week

Quantitative analysis of the defect was performed according to CT values. At 6-week, both groups could form a small amount of new bone which was immature and poorly organized. But there still was larger low-density area in the middle of ASCs group compared with BMSCs groups; 12-week, the bone formation of both increased and the central region exhibited a large number of new bone ingrowth and bone fusion. BMSC demonstrated a denser newly formed bone and tended to completely repair the defect area, while the more fragmented bone and interspersed with low-density tissue (fibrous tissue) were observed in ASCs group. However, in either group, there was still some unabsorbed bone powder in the defect area (Fig. 6a). The histomorphometric quantitative analysis demonstrated that the Tb.Th was greater in BMSCs than ASCs group in the early (6-week), but later (12-week) this gap narrowed close to equalize gradually. The BV/TV in 6 and 12 weeks were significantly greater in BMSCs-Bioss complex than the ASCs-Bioss complex (P < 0.05; Fig. 6b-d). However, Tb.Sp showed the opposite trend.

H&E staining showed that a number of encapsulated bone powder particles could be seen in the defects of two groups at 6-week. There was obvious new bone at the edge of the defect in BMSCs group, and the fibrous tissue in the middle was uniformly dense. The margins on both-side new bone were significantly shorter than that in ASCs group. By contrast, the defect in ASCs group was still filled with bundles of fibrous tissue. At 12-week, the amount and volume of bone powder in the two groups decreased. The new bone tissue at the edges grew to the center and gradually matured. Bundle fibers could be seen in the middle of the defect in ASCs group, while the defect in BMSCs group was significantly reduced, tending to heal, and mature bone tissue could be formed at both edges (Fig. 6e).

## Discussion

Impaired osteogenesis in T2DM patients leading to an overall deterioration in bone quality and structure [19–21]. In recent years, the application of stem cells has achieved unprecedented outcomes in tissue engineering to improve the osteogenic ability of T2DM [22]. However, most of studies are based on acquisition of allogeneic stem cells, which is limited in clinical practice and controversial in immunogenicity. Meanwhile, recent researches indicate that allo-MSCs will likely always activate and/or expresse MHC class II (antigens targets for rejection of grafts) in vivo at inflammation sites [11]. T2DM is a chronic inflammatory disease in nature, which means that allogeneic stem cell therapy for T2DM may face the threat of potential anti-donor response. Moreover, it also demonstrates that MSCs may lose low immunogenicity and be recognized even attacked by the immune system after continuous passaging [23]. Given all the above, autologous stem cells become more promising due to long-term transplant survival and tolerance and clinical availability without ethical issues [24]. Among them, BMSCs and ASCs are most widely used and achieve clinical outcomes [18, 25]. However, abnormal bodily condition of T2DM may suppress the BMSCs number and function, specifically, less proliferation and survivability [12, 13], and lower osteogenic differentiation [14], which limits its therapeutic potential. Furthermore, BMSCs have been proved with low availability, invasive and painful procedures for obtainment [26]. By contrast, the amount and growth curve of ASCs from T2DM were comparable to control group [15]. However, none focus on the comparison of autologous ASCs and BMSCs in T2DM patients and find out differences to choose the best fit for tissue regeneration.

We designed this study to establish T2DM models and extract ASCs and BMSCs to compare them. But, if we comply with the application of autologous stem cell that needs early extraction, intermediate detection and later implantation of extracted cells for the same rat, the difficulty to operate and the death risk of models are higher. Therefore, we extracted stem cells from allogeneic rats to clarify the influence of T2DM environment on stem cells, and finally extended this effect of T2DM on autologous stem cells. We induced T2DM rat models through a combining high-fat and high-carbohydrate diet with low-dose STZ intraperitoneal injection [18, 27, 28]. Then, ASCs and BMSCs were extracted respectively from T2DM rats. Thereupon, a batch of approaches were employed to investigate cell viability and differentiation potential. Among them, cell proliferation was closely related to the therapeutic effect of MSCs on wound closure. It was found that both cells had the ability of self-renewal and could maintain a good proliferation activity in a certain passage. In addition, clonogenicity is one of crucial properties of MSC reflecting number and amplification ability [29]. We developed a limiting dilution assay to estimate the clone formation rate and found that both them could form a large number of colony-forming units, and maintain consistency in morphology. Nonetheless, both results indicated that ASCs had better proliferation potential and viability. These were consistent with a study by Dmitrieva et al., which suggested that ASCs were more adapt to standard culture environment and easy to expansion. In contrast, BMSCs started to show significant signs of senescence after P3-4, while most ASCs did not show senescence up to P6–8 [29].

Compared with traditional cell suspension, cell-sheet is a new technique to improve the therapeutic effect of stem cells, which could highly concentrate cells, retain intercellular junction proteins, cell surface receptors and ion channels, in the meanwhile, maintain cell homeostasis and microenvironment maximally. In addition, it possesses certain mechanical strength and cell layers, retains complete cell membrane and extracellular matrix [30]. Yu miao et al. [18] constructed tissue-engineered implants by combining BMSCs sheets with implants, and results showed that the composite implants could significantly improve bone formation and deposition around implants. In order to compare the sheet-forming ability of ASCs and BMSCs from T2DM, we cultured both samples with induction medium in vitro and compared the morphology and thickness from their surface and cross-section. The Electron Microscopy and H&E staining showed that there were few differences in surface morphology, while the layers and thickness of ASCs sheets were significantly higher than that of BMSCs. It may be related to the better proliferation capacity of ASCs, or ASCs could form more extracellular matrix in vitro and thus increase the sheet thickness.

Multidirectional differentiation allows us to evaluate the differentiation potential of stem cells either into adipocytes or osteoblasts. The oil red staining demonstrated that both types of cells could be directed to form lipid droplets. ALP and alizarin red staining were used to assess early and late osteogenesis of stem cells respectively. It appeared that BMSCs could express more ALP and calcium nodules suggesting that BMSCs had a more significant osteogenic ability. In order to further compare the osteogenic abilities, we verified molecular and animal experiments. The results showed that the expression of genes and proteins related to bone formation in BMSCs were significantly higher than ASCs, although it was not obvious in the specific one. Results in vivo exhibited that the mixture of BMSCs and bone powder showed greater potential in repairing skull defects. Although its proliferation ability was inferior in the early experiment, its excellent osteogenic ability had compensated the gap, which may be associated with its more significant mineralization capacity of the matrix.

ASCs are easy availability and cause little pain for patients [31]. ASCs from T2DM have better proliferation than BMSCs, which may be caused by the early aging of BMSCs due to the low oxygen environment in bone marrow [29]. BMSCs have a better osteogenic ability, which implies that BMSCs are a preferential choice employed to escalate osteogenesis. But in the field of regeneration, cell proliferation is a key part to promote wound healing and tissue regeneration. This is due to obtaining a therapeutic dose of auto-MSCs generally require several weeks after expansion in vitro. Namely, promoting proliferation ability should be considered as a potential strategy to activate the endogenous stem cells [32]. On the other hand, for the treatment of T2DM complications, ASCs are more adaptable to the standard environment and can form more stable features in vitro [29]. In the meanwhile, Liu et al. [33] reported that human ASCs, compared with human BMSCs, had the most pronounced re-epithelialization, thickest granulation tissue and the greatest effect on human dermal fibroblast migration. We recognized that both autologous stem cells from T2DM possess general characteristics of stem cells and their own advantages. It also remains to be seen whether BMSCS or ASCs can be applied directly or modified to enhance the proliferation or osteogenesis. Of course, this study only discussed the proliferation and osteogenic ability, and didn’t cover a broader field, such as indicators to promote wound healing or chondrogenesis, which is something we need to explore further. The available data, however, demonstrate that specific and dominated properties of MSC should be taken into account when choosing and developing MSCs-related therapy to achieve the best matching and improve clinical outcomes.

## Conclusions

Compared with ASCs, BMSCs from T2DM rats have better osteogenic potential in promoting bone formation and repairing bone defects. By contrast, ASCs possess more prominent proliferative and colony-forming ability and are superior in other applications. It requires us to select or modify stem cells according to the actual situation and their respective characteristics to achieve the best therapeutic effect.

## Data Availability

The datasets used and/or analysed during the current study are available from the corresponding author on reasonable request.

## Abbreviations

ASCs: Adipose-derived stem cells
ALP: Alkaline phosphatase
auto-MSCs: Autologous mesenchymal stem cells
allo-MSCs: Allogeneic mesenchymal stem cells
BMSCs: Bone marrow mesenchymal stem cell
CCK-8: Cell counting kit-8
ECM: Extracellular matrixc
HE: Hematoxylin–eosin staining
PBS: Phosphate buffer saline
qPCR: Quantitative real time polymerase chain reaction
SEM: Scanning electron microscope
T2DM: Type 2 diabetes mellitus
